# Supplementary material for: The probability of edge existence due to node degree: a baseline for network-based predictions
Source: bioRxiv. 2023 Jan 6:2023.01.05.522939. Preprint. [Version 1] doi: 10.1101/2023.01.05.522939 (PMC9881952; doi:10.1101/2023.01.05.522939)
Supplement: Supplement 1 [file NIHPP2023.01.05.522939v1-supplement-1.pdf]

Supplemental information

XSwap parameter settings for network types

Table 1: Applications of the modified XSwap algorithm to various network types with appropriate parameter choices. For simple networks, each node's degree is preserved. For bipartite networks, each node's number of connections to the other part is preserved, and the partite sets (node class memberships) are preserved. For directed networks, each nodes' in- and out-degrees are preserved, though parameter choices depend on the network being permuted. Some directed networks can include antiparallel edges or loops while others do not.

Network type	Degree preserved	Figure	`allow_antiparallel`	`allow_loops`
simple	all	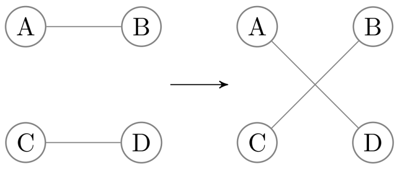	False	False
directed	in/out	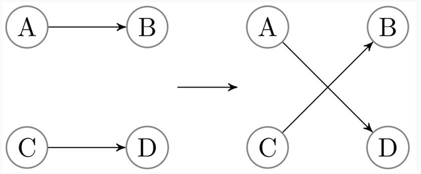	Depends on networks	Depends on networks
bipartite	Depends on directedness	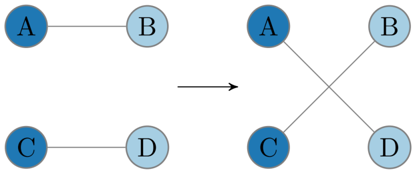	True	True

Performance of the XSwap algorithm

The performance of the XSwap algorithm depends on a number of network properties. We define network density to be the number of edges divided by the number of potential edges. Increasing network density lowers the asymptotic fraction of edges changed, as greater density prevents the algorithm from removing certain edges. Random graphs generated with a preferential attachment mechanism (via Barabási–Albert) can have a lower fraction of their edges swapped, asymptotically, as compared to uniform random graphs (via Erdős–Rényi).

bioRxiv preprint doi: https://doi.org/10.1101/2023.01.05.522939; this version posted January 6, 2023. The copyright holder for this preprint (which was not certified by peer review) is the author/funder, who has granted bioRxiv a license to display the preprint in perpetuity. It is made available under aCC-BY 4.0 International license.

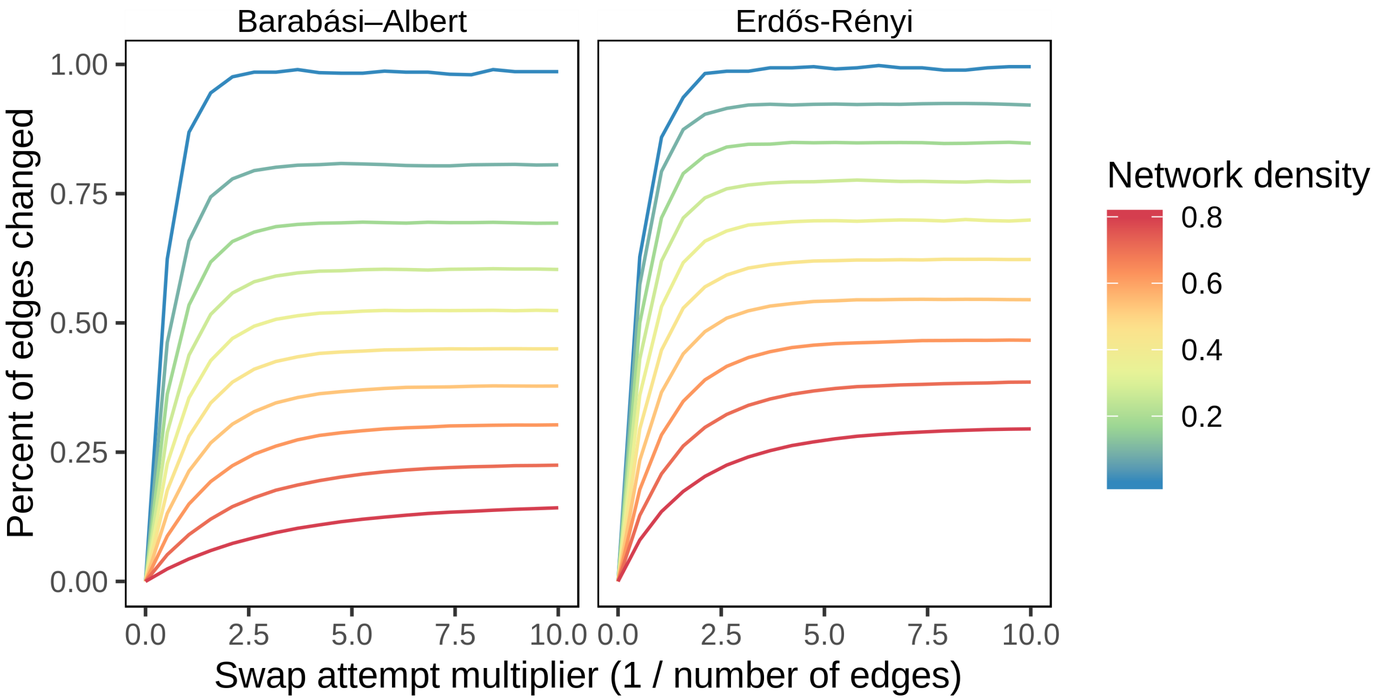

Figure 9: Higher density networks have lower asymptotic fractions of edges swapped and take more attempts to reach these values. The Barabási–Albert model produces scale-free random graphs, while Erdős–Rényi generates random graphs where all edges are equally likely.

Approximate edge prior

To approximate the edge prior, we began by making two simplifications. First, we assumed independence between node pairs. This assumption does not actually hold for the XSwap algorithm, though it is a reasonable simplification for large, sparse networks. Second, we assumed that the XSwap process is stationary. This assumption also does not actually hold, but it was made because it significantly simplifies the problem. A single node pair has two possible states, "edge" and "no edge". These states are not transient, and they are not periodic so long as more than one possible swap exists in the network. In almost all cases, then, our simplified model of the algorithm gives the state of a node pair as an ergodic process, independent of other node pairs.

Let $A_{i,j}$ represent the existence of edge (i, j) For a given node pair, (i, j), then, let $q_{i,j}$ represent the transition probability from the "no edge" state to the "edge" state in one successful iteration of the XSwap algorithm. Let $r_{i,j}$ represent the probability of the opposite transition ("edge" to "no edge") in one successful iteration. With "no edge" represented as $[1, 0]^T$ and "edge" represented as $[0, 1]^T$, the transition matrix, P, is given by the following:

$$P^T = \begin{bmatrix} 1-q & r \\ q & 1-r \end{bmatrix}$$

The stationary distribution of this system should correspond to the distribution when the number of swaps goes to infinity. It can be found by computing the eigenvectors of the system, as we know that the stationary distribution vector, $\mathbf{v}$ satisfies $P^T\mathbf{v} = \mathbf{v}$. The eigenvector $\mathbf{v}$, normalized to sum to 1 as a probability vector, is given by

$$\mathbf{v} = \frac{1}{r+q}\begin{bmatrix} r \\ q \end{bmatrix}$$

The asymptotic edge probability is therefore

bioRxiv preprint doi: https://doi.org/10.1101/2023.01.05.522939; this version posted January 6, 2023. The copyright holder for this preprint (which was not certified by peer review) is the author/funder, who has granted bioRxiv a license to display the preprint in perpetuity. It is made available under aCC-BY 4.0 International license.

$$\frac{q}{r+q}.$$

Since node pairs are being treated as independent, the probability of an edge being created in one successful iteration, given that the edge does not currently exist, is the ratio of the number of edge choices involving nodes i and j to the total number of possible swaps, S. Let $d(u_i)$ represent the degree of source node i and $d(v_j)$ represent the degree of target node j.

$$q_{i,j} = \frac{d(u_i)d(v_j)}{S}$$

Similarly, the probability of an edge being eliminated in one iteration is the ratio of the number of edge choices involving (i, j) and any other valid edge to the total number of possible swaps. Let m be the total number of edges in the network.

$$r_{i,j} = \frac{m - d(u_i) - d(v_j) + 1}{S}$$

The approximate edge prior is, therefore,

$$\frac{d(u_i)d(v_j)}{m - d(u_i) - d(v_j) + 1 + d(u_i)d(v_j)}.$$

Unfortunately, we found that the above edge prior approximation is a poor approximation in many cases. We found that the following modified form (introduced in Methods) affords a superior approximation:

$$P_{i,j} = \frac{d(u_i)d(v_j)}{\sqrt{(d(u_i)d(v_j))^2 + (m - d(u_i) - d(v_j) + 1)^2}}$$

Interestingly, this expression can be derived by normalizing the eigenvector $\mathbf{v}$ to be a unit vector in the 2-norm instead of the 1-norm; that is, we use the value $q/\sqrt{r^2 + q^2}$ instead of $q/(r+q)$. Because the modified form of the approximation offers a much superior fit to the data, we chose to include only the modified version in the released Python package, and we used the modified form throughout our analysis.

Networks used for comparison

Data	Network	Nodes	Edges
Hetionet	AdG	Source: 402, Target: 20945	102240
	AeG	Source: 402, Target: 20945	526407
	AlD	Source: 402, Target: 137	3602
	AuG	Source: 402, Target: 20945	97848
	BPpG	Source: 11381, Target: 20945	559504

bioRxiv preprint doi: https://doi.org/10.1101/2023.01.05.522939; this version posted January 6, 2023. The copyright holder for this preprint (which was not certified by peer review) is the author/funder, who has granted bioRxiv a license to display the preprint in perpetuity. It is made available under aCC-BY 4.0 International license.

	CCpG	Source: 1391, Target: 20945	73566
	CbG	Source: 1552, Target: 20945	11571
	CcSE	Source: 1552, Target: 5734	138944
	CdG	Source: 1552, Target: 20945	21102
	CrC	1552	6486
	CuG	Source: 1552, Target: 20945	18756
	DaG	Source: 137, Target: 20945	12623
	DdG	Source: 137, Target: 20945	7623
	DpS	Source: 137, Target: 438	3357
	DuG	Source: 137, Target: 20945	7731
	GuG	20945	265672
	GcG	20945	61690
	GiG	20945	147164
	GpMF	Source: 20945, Target: 2884	97222
	GpPW	Source: 20945, Target: 1822	84372
PPI	Sampled	3992	255522
	Literature	3992	364743
	Systematic	3916	12913
bioRxiv	Sampled	4587	30686
	<2018	4615	43691
	All time	4615	44963
TF-TG	Sampled	Source: 142, Target: 1396	2689
	Literature	Source: 144, Target: 1406	3496
	Systematic	Source: 144, Target: 1417	29177

Edge prediction features

In the table that follows, let $k(u)$ denote the set of neighbors of node u. Let $\mathbf{A}$ represent the normalized Laplacian adjacency matrix, and let y_u be a vector with all ones except for a one in the u-

bioRxiv preprint doi: https://doi.org/10.1101/2023.01.05.522939; this version posted January 6, 2023. The copyright holder for this preprint (which was not certified by peer review) is the author/funder, who has granted bioRxiv a license to display the preprint in perpetuity. It is made available under aCC-BY 4.0 International license.

th position. x For a directed graph, let $A(u)$ denote the set of nodes that node u points to and $D(u)$ the set of nodes that point to u. All definitions that follow are the score between nodes u and v.

Table 2: Edge prediction features.

Feature	Definition	Citation
Jaccard index	$\frac{\|k(u)\cap k(v)\|}{\|k(u)\cup k(v)\|}$	[28]
Preferential attachment score	$\|k(u)\|\|k(v)\|$	[28]
Resource allocation index	$\sum_{w\in k(u)\cap k(v)} \frac{1}{\|k(w)\|}$	[26]
Adamic/Adar index	$\sum_{w\in k(u)\cap k(v)} \frac{1}{log\|k(w)\|}$	[29]
Random walk with restart score	$c\left[\left(\mathbb{I}-(1-c)\mathbf{A}\right)^{-1}\mathbf{y}_u\right]_v$	[30,31]
Inference score	$\frac{\|A(u)\cap D(v)\|}{\|A(u)\|}+\frac{\|D(u)\cap D(v)\|}{\|D(u)\|}$	[32]
